# Development of a Human Activity Recognition System for Ballet Tasks

**DOI:** 10.1186/s40798-020-0237-5

**Published:** 2020-02-07

**Authors:** Danica Hendry, Kevin Chai, Amity Campbell, Luke Hopper, Peter O’Sullivan, Leon Straker

**Affiliations:** 1 School of Physiotherapy and Exercise Science, Curtin University, Perth, Western Australia Australia; 2 Curtin Institute for Computations, Curtin University, Perth, Western Australia Australia; 3 Western Australian Academy of Performing Arts, Perth, Western Australia Australia

## Abstract

**Background:**

Accurate and detailed measurement of a dancer’s training volume is a key requirement to understanding the relationship between a dancer’s pain and training volume. Currently, no system capable of quantifying a dancer’s training volume, with respect to specific movement activities, exists. The application of machine learning models to wearable sensor data for human activity recognition in sport has previously been applied to cricket, tennis and rugby. Thus, the purpose of this study was to develop a human activity recognition system using wearable sensor data to accurately identify key ballet movements (jumping and lifting the leg). Our primary objective was to determine if machine learning can accurately identify key ballet movements during dance training. The secondary objective was to determine the influence of the location and number of sensors on accuracy.

**Results:**

Convolutional neural networks were applied to develop two models for every combination of six sensors (6, 5, 4, 3, etc.) with and without the inclusion of transition movements. At the first level of classification, including data from all sensors, without transitions, the model performed with 97.8% accuracy. The degree of accuracy reduced at the second (83.0%) and third (75.1%) levels of classification. The degree of accuracy reduced with inclusion of transitions, reduction in the number of sensors and various sensor combinations.

**Conclusion:**

The models developed were robust enough to identify jumping and leg lifting tasks in real-world exposures in dancers. The system provides a novel method for measuring dancer training volume through quantification of specific movement tasks. Such a system can be used to further understand the relationship between dancers’ pain and training volume and for athlete monitoring systems. Further, this provides a proof of concept which can be easily translated to other lower limb dominant sporting activities

## Key Points

- Deep learning models were shown to have acceptable accuracy when applied to recognised ballet-specific jumping and leg lifting tasks in a population of 23 dancers.
- A system of multiple sensors (six per dancer) was shown to have the greatest accuracy; however, the optimal single sensor model also performed with acceptable accuracy.
- The inclusion of all six sensors yielded the highest degree of accuracy: however, fewer sensors still provided an acceptable degree of accuracy. For real-world application, minimal sensors are required to reduce athlete burden.
- The method demonstrated for model development is highly translatable for future developments in other lower limb dominant sporting activities.

## Introduction

The quantification of training volumes in sport has significantly advanced knowledge regarding the development of musculoskeletal pain disorders in athletes [1]. Due to a high prevalence of lower limb and lower back pain and associated disability in dancers, there is a growing body of literature focussing on physical training volume in this population [2–4]. Assessment of dancer training volumes has been largely derived from subjective, self-reported measures such as schedules and activity diaries [2, 4], which are imprecise and are frequently biased [5]. Furthermore, these methods are limited to the number of hours of training/performing and do not account for individual dancer training volume or specific movements. In quantifying training volume, specific movements likely to be provocative of pain should be considered [6], such as jumping and landing, which has been associated with development of foot/ankle, knee and lower back pain [7, 8], and lifting the leg to the front, side or behind the body, which has been associated with hip and lower back pain [9]. Accurate and detailed measurement of a dancer’s training volume is a key requirement in understanding the relationship between training volume and pain disorders. However, no automated and objective system exists which provides the sensitivity to measure the training volume of specific movements performed by individual dancers.

Small, relatively inexpensive, commercially available wearable sensors have been rapidly adopted in mainstream sports for the objective quantification of training volume [5]. Sensor units typically incorporate accelerometry technology to evaluate movement magnitudes and provide an estimation of metabolic demands of sporting activities [5]. Specific movement tasks may be better detected using inertial measurement units (IMU), which incorporate accelerometers, gyroscopes and magnetometers allowing for the use of multiple sensor outputs to identify specific movement tasks [10]. Accelerometers measure the rate of change of velocity via linear accelerations, and gyroscopes measure orientation and angular velocity [11]. Magnetometers provide directional information, similar to a compass, by measuring magnetic field strength [11].

Machine learning algorithms, when applied to IMU data, have provided new insight into the evaluation of athletic movement demands through the automatic recognition of sport-specific movements, ‘human activity recognition’ (HAR) [12]. Machine learning algorithms learn from data and can perform better than manually hard-coded rules for complex problems. For example, machine learning algorithms have been applied to data from a single wrist-worn IMU in tennis, demonstrating an accuracy of 97.4% when classifying three different tennis strikes [13]. Accuracy reduced to 93.2% when nine different types of tennis strikes were included in the algorithm [13], suggesting that machine learning performance reduces with greater levels of feature classification. Further, a manufacturer developed algorithm for detecting jumps during volleyball using a sacrum mounted sensor, with an average precision (accurate detection of relevant events) and recall (accurate rejection of irrelevant events) of 99.8% and 87.9%, respectively [14], as well as with excellent specificity and sensitivity, correctly identifying 96.8% of the jumping activities and 100% of non-jumping activities, with no false negatives [15]. These results suggest that there is great potential for HAR using IMU in dance to provide specific automated means of quantifying dance-specific movements.

Recently, more sophisticated machine learning techniques have been developed, such as deep learning for HAR [16, 17]. Deep learning models are able to automatically learn features from raw data and are often able to achieve better performance than traditional machine learning because their added complexity allows the models to take greater advantage of larger and more complex training datasets [16]. A convolutional neural network (CNN) is a deep learning technique commonly used for image classification and object detection and can be applied to any type of ordered data such as wearable sensor data (time series) for HAR [16].

The placement and number of sensors utilised can influence accuracy of HAR [18]. Within HAR, the inclusion of multiple sensors at specific locations can impact the accuracy of classification, as well as the variety of activities that can be detected [18]. However, wearing multiple sensors is burdensome for the athlete. As a result, researchers aim to achieve a minimum number of sensors while still developing HAR models with the highest possible degree of accuracy [18].

Ballet is an art form founded by a number of specific movement activities. Repeated jumping and leg lifting tasks are common ballet movements that have been associated with the development of pain disorders [19, 20]. Within a single ballet class, dancers can perform over 200 jumps, with a large variety of biomechanical demands and over half of which land unilaterally [20]. Similarly, dancers may lift their leg to the front, side or behind the body and the speed and pathway of the leg movement depends upon the specific activity they are performing [21, 22]. Finally, activities in ballet are rarely performed in isolation; instead, they are dictated by their preceding and proceeding movements, which can be termed transitions. Currently, it is unclear as to whether transitions have been incorporated into HAR models for sporting activities. However, when applied to ballet, a HAR model needs to recognise specific activities while also accounting for the large, within activity variations and consider transitions.

While there is a growing body of literature supporting the use of machine learning for activity recognition in sports [12, 17], based on review of the literature, to our knowledge, there are no reports of a machine learning approach to assist in quantifying ballet specific movement tasks. Thus, the purpose of this study was to develop a HAR system using wearable sensor data to accurately identify key ballet movements (jumping and lifting the leg), allowing for objective quantification of training volume in ballet. Our primary objective was to determine if machine learning can accurately identify key ballet movements during dance training. The secondary objective was to determine the influence of the location and number of sensors on accuracy.

## Methods

### Participants

We recruited 23 female pre-professional dancers (mean (SD) age, 19.6 (1.2) years) from a university dance institution. Dancers were included in the study if they were currently enrolled in one of the full-time vocational dance training programmes at the institution, uninjured at the time of data collection and were participating in a minimum of 8 hours of ballet training per week. Only female dancers were recruited for this study as the movement profile of females and males are different in ballet, where many dance movements are gender specific, and there are differences in the biomechanics demonstrated between males and females [23, 24]. Additionally, there is greater female participation at a pre-professional level. Dancers were excluded from the study if they were currently injured or unwell. This study was approved by the university’s human research ethical committee (HRE2017-0185) with reciprocal ethical approval from the dance institution. Informed consent was obtained from all individual participants included in the study.

### Data Collection and Tasks

Data collection took place in groups of 2 to 5 dancers within a standard ballet studio, equipped with a common sprung dance studio floor. Following a self-directed warm-up and attachment of sensors, dancers performed a series of discrete movement tasks commonly performed within classical ballet, jumping and leg lifting tasks (see Tables 1 and 2), i.e. the tasks were performed in isolation rather than embedded within a choreographed sequence. The jumping and leg lifting tasks were selected to reflect the movement sequences performed within a typical ballet class and were performed in the same order by all dancers. Jumping tasks incorporated small jumps and large jumps, landing bilaterally and unilaterally, on the right and left leg. The leg lifting tasks were performed to the front, side and behind the body, on the right and left leg*.* To allow for movement variability between the tasks, timing, magnitude and arm movements for the discrete movement tasks were determined by the dancers, reflecting normal practice. These tasks were then performed within specified choreographed sequences and to music, typical of a normal ballet class. The discrete tasks, including the order they were performed in, and examples of choreographed sequences are detailed in Table 2. Data collection for each dancer took approximately 45 min.

**Table 1:** Levels of classification for movement tasks

Jumping tasks: levels of classification		
Movement (1)	Jump type (2)	Laterality (landing leg) (3)
Jump	Bilateral landing small jump	Bilateral
	Unilateral landing small jump	Right
		Left
	Unilateral landing large jump (leap)	Right
		Left
Leg lifting task: levels of classification		
Movement (1)	Direction of leg lift (2)	Laterality lifted leg (3)
Leg lift	Front	Right
		Left
	Side	Right
		Left
	Back	Right
		Left
Other—used only for models when transitions included

**Table 2:** Order and description of discrete ballet movement tasks and example of choreographed sequences

Ballet movement	Description
Leg lifting tasks	
Grands battements (devant, a la seconde, derriere)	In a controlled, large amplitude tossing or throwing action, the dancer flexes at the hip to bring the lower limb with the knee held in extension to the front of the body 3 times in succession closing into fifth position each time. The dancer then repeats this movement to the side of the body and then behind the body (hip and lumbar spine extension). This is repeated on the other leg.
Develloppe (devant, a la seconde, derriere)	In a slow, controlled unfolding movement, the dancer lifts the leg to the front of the body. This is repeated to the side and the back. This is repeated on the other leg. This is repeated 3 times.
Battement Lente (devant, a la seconde, derriere)	In a slow, controlled movement, the dancer lifts the leg to the front of the body, maintaining knee extension. This is repeated to the side and the back. This is repeated on the other leg. This is repeated 3 times.
Jumping tasks	
Sauté in first position	The dancer commences in first position of the feet (lower limbs externally rotated and heels placed together) and performs 8 vertical jumps landing bilaterally.
Changement in fifth position	The dancer commences in fifth position of the feet (lower limbs externally rotated and feet crossed) and performs 8 vertical jumps changing the front foot upon landing.
Entrechat Quatre	The dancer commences in fifth position of the feet (lower limbs externally rotated and feet crossed) and performs 4 vertical jumps beating the legs in air before landing bilaterally with the same foot in front. This was performed with the right leg and left leg starting in front.
Assemblé	The dancer commences in fifth position and swishes one leg out to the side as they take off, they gather the legs in the air together and land before immediately taking off for the next jump. This is repeated 6 times.
Jeté ordinaire	The dancer commences in fifth position and swishes one leg out to the side as they take off, they then land on the limb that they swished to the side. This is repeated 8 times.
Temps levé	A single leg vertical jump and land performed 5 times in succession
Grand Jeté en avant	A big leap. To prepare for the movement, the dancer performed a travelling sequence to generate momentum, as they would normally do within a dance class. This was repeated 2 times on each leg.
Grand Jeté en tournant	A big leap turning the body in the air. This was repeated 3 times on each leg
Choreographed sequence example	
Slow leg lift sequence	**Develloppe devant with right leg**, *lower the leg to pass through first position to***lift into battement lente derriere**. *Lower the leg into fifth position.* **Develloppe the left leg a la seconde**. *Carry the leg, still lifted to derriere.***Hold the leg lift derriere** and *pivot the body slowly 360°.* *Once returned to original positon, close in 5th position. Travelling step into a pirouette.*
Jump sequence	*Travelling step to the right***, jeté ordinare to the right, temps levee** *Travelling step to the left***, jeté ordinare to the left, temps levee** *Travelling step to the right***, jeté ordinare to the right, temps levee** *Travelling step to the left***, assemble** *Remaining on the floor rise up on toes from bent knee position***. Three changements***changing direction on each on to turn 360°*

Bold indicates movements for classification and italics indicate transition movement

### Instrumentation/Sensors and Video

**Fig. 1:**
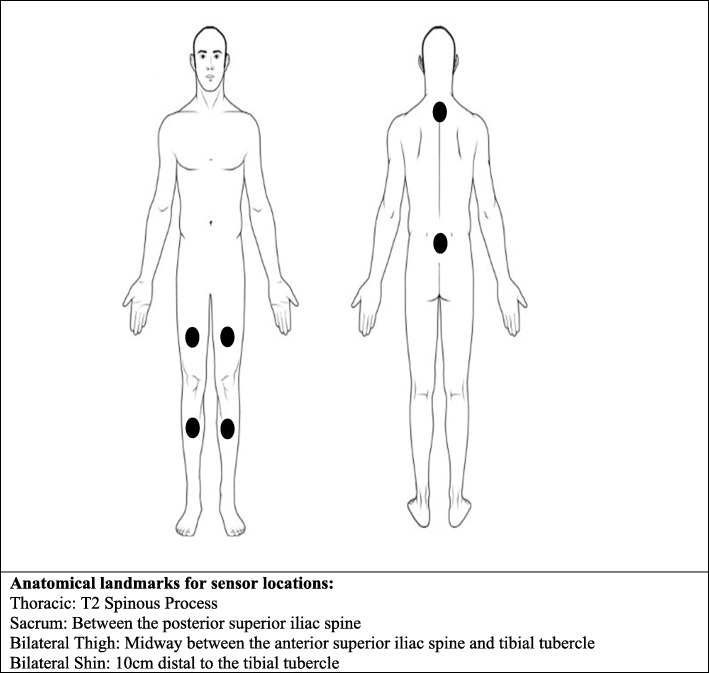
Wearable sensor locations

Dancers wore six ActiGraph Link wearable sensors (ActiGraph Corporation, Pensacola, FL), operating at 100 Hz and with the gyroscope and magnetometer enabled. The Actigraph Link is a small commercially available triaxial wearable sensor which integrates data from an on-board accelerometer, gyroscope and magnetometer. The ActiGraph sensors were secured to the skin using a double-sided tape and a single piece of hypoallergenic tape covering at the anatomical locations as shown in Fig. 1. Sensors were placed on the thoracic spine (used in previous sporting activity recognition research [25–27]), sacrum (recommended as this is close to an individual’s centre of mass [18]) and lower limbs (to capture lower limb movement). On the lower limbs, sensors were placed bilaterally in order to detect the different asymmetrical movements of dance. Both thigh and shin sensors were placed on each lower limb as the shin would likely provide a larger amplitude of acceleration due to the larger axis of rotation (particularly in leg lift tasks), thus providing different information for the HAR model development. Additionally, dancers were simultaneously video recorded using a GoPro Session 5 (GoPro. Inc., USA), capturing 100 frames per second.

### HAR System Development

The process of developing the HAR system is described in detail below [28].

#### Data Preparation

Following data collection, the ActiLife software (version 6.13.3) was used to output date-time stamped files of each wearable sensor’s raw data including triaxial accelerometer, gyroscope and magnetometer outputs.

The video data was manually annotated frame by frame by a ballet expert to identify and classify the specific movements at 3 levels (see Table 1). The first level of classification determined if the dancer was performing a jump or a leg lifting task. At the second level of classification, jumps were identified based upon size (smaller jumps or large leaps) and whether they landed bilaterally or unilaterally. Smaller jumps included both bilateral and unilateral landings, whereas all large leaps land unilaterally. At the second level of classification, leg lifting tasks were classified by the direction (front, side or back). The third level of classification described laterality of the tasks, i.e. whether the dancer was landing on the right or left leg during unilateral jumping tasks and whether they were lifting their right leg or left leg during leg lifting tasks. Movements that dancers performed that were not these specific movements were left without annotation and considered ‘other’ at all 3 levels of classification.

A customised LabVIEW program (LabVIEW 2017 SP1, National Instruments, Austin, TX, USA) was used to synchronise and merge the six sensor files with the video-based specific movement annotation file. Time synchronisation was based on a standardised movement; dancers were instructed to stand still for 5 s, then perform a double leg heel raise and then stand still for another 5 s at the beginning of data collection. This generated an accelerometry signal which was similar on all sensors, with a period of stillness on either side which could be used for visual synchronisation with the video data. Following synchronisation, unwanted data was removed. Unwanted data were time periods where dancers were not performing the discrete movement tasks or choreographed sequences of movements. This included periods such as breaks, when dancers were being instructed on what movements to perform, as well as short practice sessions performed by the dancers.

#### Segmentation

The data was segmented at a fixed window size of 100 frames to align with the 100-Hz sensor and 100-fps video data, resulting in the dataset being split into 1-s segments of data. Additionally, overlapping segments were created in order to capture enough data for detecting events near the window boundaries. An overlap size of 75% was used as it achieved better results compared with other sizes (0%, 25% and 50% were tested).

#### Feature Extraction

Initial experimentation was performed, extracting a number of time and frequency domain features commonly used in HAR with wearable sensors [10, 29, 30], such as calculating the average and median signal values for various time segments and discrete cosine transforms. These features were used with a number of machine learning approaches including, but not limited to, logistic regression, random forests, support vector machines and shallow neural networks. However, these approaches did not achieve satisfactory results. CNNs were therefore used to learn and extract features automatically from the dataset [16].

#### Feature Selection

Exhaustive feature selection was applied in order to evaluate all location combinations of sensors for training our models.

#### Classification

A number of CNN architectures were experimented with, using different numbers of layers, filters, filter sizes, activation functions and combinations of convolution and pooling layers. The filter size (layer 1, 25 horizontal, 9 vertical; layer 2, 10 horizontal, 9 vertical) for the convolution layers was selected to allow for filters to learn for each sensor location at a time, i.e. filters to be learnt for the left shin *x*, *y* and *z* along with the accelerometer, gyroscope and magnetometer all at once and then the next sensor location would be learnt. The optimisation algorithm applied to the entire model was the adaptive momentum (Adam) algorithm [31]. Further detail on model architecture can be seen in Additional file 1.

Two models were developed for each possible sensor combination, first without the consideration of transition movements and the second with the consideration of transition movements. Data that was annotated as ‘other’ was considered transition movement.

### Determining Model Performance/Statistical Testing

The performances of the models were evaluated using a leave-one-out cross-validation method [30]. In the leave-one-out cross-validation, the classification model is trained on data from all of the participants except one, which is ‘held out’ and used as the test dataset. The process is repeated until all participants have served as the test data, and the performance evaluation results are averaged [30].

**Fig. 2:**
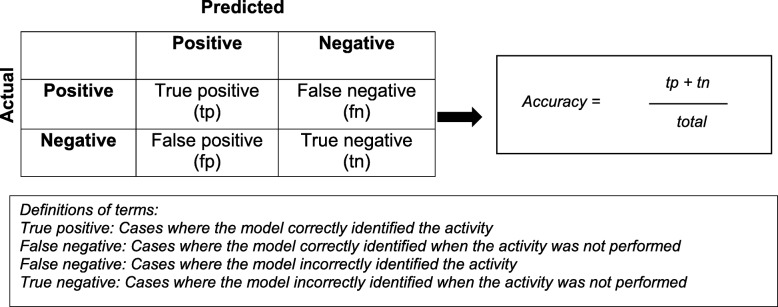
Components of a confusion matrix

To explore the primary aim, determining the performance of the model in detecting the movement tasks, the models were evaluated using all six sensors, at each of the three levels of classification. The models developed without consideration of transition movements allowed comparisons with existing literature, while the addition of transitions allows for greater ecological validity [32]. To explore the secondary aim, determining to what extent the number and location of sensors affect performance of the model, the model was evaluated using all other possible sensor combinations (i.e. all possible combinations for five sensors, four sensors, three sensors, etc.) at each of the three levels of classification. This allowed determination of the best combination for each number of sensors. To interpret the performance of the models, confusion matrices were constructed for each participant with every combination of sensors and averaged across the population. The components of a confusion matrix are demonstrated in Fig. 2. This was used to calculate the degree of accuracy for each model in classifying the movements at each of the three levels of classification for all sensor combinations. Accuracy was calculated by the sum of the true positive and true negative divided by the total [13].

## Results

### All Six Sensors

At the first level of classification, including all six sensors, the model without transitions performed with 97.8% accuracy. The degree of accuracy reduced at the second and third levels of classification to 83.0% and 75.1%, respectively. When transitions were included, the performance of the model reduced to 84.2% accuracy at the first level of classification, 77.1% at the second level and 73.5% at the third level.

### Different Sensor Combinations

Without transitions, the model performed with a high degree of accuracy at the first level of classification regardless of the number of sensors the dancer was wearing (see Table 3). At the second and third levels of classification, there were reductions in performance of the model with reduced sensors regardless of the sensor combination (see Table 3).

**Table 3:** Degree of accuracy for different sensor combinations at all three levels of classification—without transitions

	Level 1			Level 2			Level 3		
Number of sensors (number of possible sensor combinations)	Accuracy score mean (range)	Best	Worst	Accuracy score mean (range)	Best	Worst	Accuracy score mean (range)	Best	Worst
5 (6)	98.2% (98–98.5%)	L shin L thigh R shin R thigh Sacrum	L thigh L shin R thigh Sacrum Thoracic	81.8% (81.3–81.8%)	L shin L thigh R shin R thigh Sacrum	L shin L thigh R thigh Sacrum Thoracic	74.9% (74.1–76.3%)	L shin L thigh R shin R thigh Sacrum	L shin L thigh R thigh Sacrum Thoracic
4 (15)	98.1% (97.8–98.4%)	L shin L thigh R shin R thigh	L thigh R shin R thigh Sacrum	81.3% (79.3–82.4%)	L shin R shin R thigh Sacrum	L shin L thigh Sacrum Thoracic	73.8% (71.8–75.1%)	L shin R shin R thigh Sacrum	R shin R thigh Sacrum Thoracic
3 (20)	98% (97.6–98.2%)	L shin R thigh Sacrum	R shin Sacrum Thoracic	79.5% (73.7–81.7%)	L shin R shin Sacrum	L shin L thigh Thoracic	72.0% (65.2–74.5%)	L shin R thigh Sacrum	L shin L thigh Thoracic
2 (15)	97.7% (97.2–98.1%)	L shin R thigh	Sacrum Thoracic	75.8% (69.7–80.2%)	L shin R thigh	L shin L thigh	68.0% (61.5–72.5%)	L shin R thigh	L shin Thoracic
1 (6)	97.3% (97–97.7%)	R thigh	R shin	67.1% (60.2–76.5%)	Sacrum	Thoracic	56.5% (38.0–65.3%)	Sacrum	Thoracic

A similar trend existed when transitions were applied (see Table 4).

**Table 4:** Degree of accuracy for different sensor combinations at all 3 levels of classification—with transitions

	Level 1			Level 2			Level 3		
Number of sensors (number of possible sensor combinations)	Accuracy score mean (range)	Best	Worst	Accuracy score mean (range)	Best	Worst	Accuracy score mean (range)	Best	Worst
5 (6)	84% (83.6–84.4%)	L shin L thigh R shin R thigh Thoracic	L thigh L shin R thigh Sacrum Thoracic	76.2% (75.9–76.6%)	L shin L thigh R shin R thigh Thoracic	L shin, L thigh R shin R thigh Sacrum	73.6% (73.2–74%)	L shin L thigh R shin R thigh Sacrum	L shin L thigh R shin R thigh Thoracic
4 (15)	83.4% (82.5–84.0%)	L shin R shin R thigh Sacrum	L shin L thigh Sacrum Thoracic	75.3% (74.5–75.9%)	L shin R shin R thigh Thoracic	L shin L thigh Sacrum Thoracic	73.0% (71.5–74%)	L shin L thigh R shin R thigh	L shin L thigh Sacrum Thoracic
3 (20)	82.9% (82.1–83.6%)	L shin R shin Thoracic	L shin L thigh Sacrum	73.9% (70–75.4%)	L shin R shin Sacrum	L shin L thigh Thoracic	71.6% (67.1–73.3%)	L shin R shin R thigh	L shin L thigh Thoracic
2 (15)	82.1% (81.2–82.9%)	L shin R high	L shin Thoracic	71.2% (67.3–74.4%)	L shin R thigh	L shin Thoracic	68.5% (64–71.8%)	L shin R thigh	L shin Thoracic
1 (6)	80.6% (78.0–81.6%)	R thigh	Thoracic	64.7% (58.5–70%)	Sacrum	Thoracic	61.0% (47.4–67%)	Sacrum	Thoracic

## Discussion

Using triaxial accelerometer, magnetometer and gyroscope outputs of six wearable sensors, a CNN model was trained to identify dance-specific jumping and leg lifting tasks at three different levels of classification. Models based on data without transitions performed superiorly to models which considered transition movements. There was a gradual reduction in model performance with increased levels of classification and performance also reduced with reduced sensor numbers and for different sensor location combinations.

At the first level of classification, determining if the dancer was jumping or lifting their leg, using all six sensors and not including transitions, the model developed in this study performed superiorly to previously developed HAR algorithms in sport [10, 12, 17, 33], with an average degree of accuracy of 98.2%. Convolutional neural networks have previously been applied to a single wearable sensor’s accelerometer output to identify 10 different specific strikes in beach volleyball at a single level of classification with a lower classification accuracy of 83.2% [33]. The results of the current study are closer to those of machine learning programmes which have been developed for the recognition of bowling tasks in cricket (99% specificity and 98.1% sensitivity) [25] and tackles in rugby (97.6% accuracy) [27]. While manufacturer-developed algorithms have been developed to detect jumping on other sporting populations with similar accuracy, these have not been validated in dance-specific jumps [14, 15]. Further, they only detect jumping movements and not activities [14]. Therefore, the current study provides a system to detect specific dance movements for training volume monitoring in dance that is as robust as that being used for movement measurement in elite sport.

As expected, the inclusion of transition movements reduced the accuracy of the model at the first level of classification (mean accuracy 84%). To our knowledge, no previously developed HAR models and algorithms have applied transition movements in the development of their models within sport. The inclusion of transitions is more ecologically valid as movement is rarely performed discretely, rather within the context of the sport or activity they are part of [32]. While the application of transitions reduced the accuracy of the model, developing a model with transitions will likely promote superior real-world performance of the system [32]. With this in mind, we contend that future system developments should include transition movements within the model development. As a result, the remainder of this discussion will reflect the results including transitions.

The degree of accuracy reduced with increasingly complex classification levels, from 84.2% at the first level, to 77.1% at the second and 73.6% at the third level. This supports previous findings of diminishing accuracy with increasing complex classifications during tennis (97.4% at level 1 and 93.2% at level 2) [13]. While there are currently no thresholds defined in terms of acceptability in degree of accuracy, a potential error rate of between 15.8% at the first level of classification and 22.9% at the second level is still superior to self-reported measures which can have errors of up to 36.9% [34].

The HAR system presented included three levels of classification, providing additional critical information that is not reflected in training schedules [2], nor in manufacturer-developed algorithms for jump detection [14, 15]. At the second level of classification, the jumping tasks were classified based upon jump size and whether the dancer landed bilaterally or unilaterally. This information may be pertinent given that during unilateral landings, the substantial ground reaction forces evident in dancers are absorbed by a single leg [20], imposing greater risk towards musculoskeletal pain development [35]. The leg lifting tasks were categorised according to leg lift direction. This might help inform musculoskeletal risk, given that repeated leg lifting tasks to the front and side of the body have implications for the development of hip pain, while repeated leg lifting tasks behind the body have implications for the development of back pain [19]. At the third level of classification, laterality was identified with jumps and leg lifts, with an accuracy of 73%. Of note, this is the first HAR system developed that includes laterality. Despite the overall decreased accuracy of the HAR with increased classification, this detailed information may provide critical insights to better understanding the relationship between training volumes and musculoskeletal pain in this population.

Our results demonstrate diminished accuracy with decreased number of sensors, particularly at the second and third levels of classification. It is likely that this was due to a greater number of potential activities that were being recognised at these levels, thus reducing the size of the dataset for each activity, and also looking at the activities in greater detail. Interestingly, the best sensor combination for 5, 4, 3 and 2 sensors all included the right thigh and left shin sensors. We believe that this is because of the, largely, lower limb dominant and asymmetrical nature of ballet movements, where bilateral sensors located in different locations would provide varying information to a HAR model. Thus in future HAR model developments, sensor location on each lower limb should be considered.

Wearing multiple sensors can be burdensome to the dancer, as well as require greater equipment, data collection and processing demands [32]. Additionally, the aesthetics of ballet focus on clean, unimpeded movements and line of the leg and torso, in both training and performance settings [36]. It is unlikely that an elite dancer or athlete would regularly wear six sensors and within other sports a single upper back worn sensor is more common [25, 27]. Our study demonstrated a single sensor worn on the upper back having the poorest accuracy. This may be due to the nature of the tasks considered which are lower limb dominant, and dancers maintain an upright posture through the thorax. Our results do however indicate that a single sensor worn on the sacrum would allow for reasonable accuracy in detecting the movement tasks of interest to this study, at the first and second levels of classification (81.5% and 70%, respectively). This may be optimal, as a single sensor on the sacrum is easily concealed providing scope for the use of the sensor system without detracting from the traditional aesthetic lines created in classical ballet, nor impeding the dancers’ movement.

### Strengths and Limitations

This system can be used to measure a dancer’s training volume with regards to multiple specific movement tasks, providing coaches, medical staff and dancers with information for training volume monitoring and implication for pain development. The accuracy achieved by the models is promising with the strengths being the dance population the models were developed on and ecological validity of the data collected. The dancers involved in the study represented a cross-section of pre-professional dancers enrolled in a university pre-professional dance programme, inclusive of both classical ballet and contemporary dance majors, thus displayed a range of differences in technical abilities. The benefit of this is that the HAR system should be generalisable to a range of pre-professional dancers with varying abilities; however, the system may not be accurate in activity recognition for either less experienced dancers or more experienced, professional dancers. Additionally, the inclusion of transition movements allows for greater real-world application of the HAR system.

This HAR system was limited to the recognition of jumping and leg lifting tasks and developed using only a female population of dancers. Further development of a system to measure training volume in dancers should include a greater variety of movement tasks such as pirouettes, pointe work and travelling phrases of movement. Such development should also include male dancers, considering specific movements that have been associated with the development of pain in male dancers, such as partnering work, lifting and jumping. As technological advances in wearable sensors continue, embedded sensors in dancers’ footwear and attire may also promote further opportunity.

While the models in the current study are developed to recognise dance-specific movement tasks, the methodology demonstrated is transferrable and generalisable for HAR of other lower limb dominant sporting activities, such as kicking in Australian football and soccer, or specific jumping tasks demonstrated during athletics and basketball. A limitation of the developed CNN model is that we are unable to determine the contribution of specific sensor types (accelerometer, magnetometer and gyroscopes) in recognising the activities. Further model development could be performed using only specific selections of the different sensor data from specific locations, for example using only magnetometer data from the sacrum sensor and gyroscope data from the thoracic sensor. However, this would involve the training and evaluation of many thousands of models. However, our results highlight the importance of the inclusion of transition movements in HAR model development and also consideration of activities at multiple levels of classification, allowing for further insight on the specific workloads that athletes are exposed to within training and competition.

## Conclusions

A HAR model developed with transition movements was robust enough to identify jumping and leg lifting ballet tasks in real-world exposures. Further, the HAR model could provide some indication of size of the jumps, whether the dancer was landing bilaterally or unilaterally and the direction that the dancer was lifting the leg. While the use of all six sensors provided the most accurate identification, fewer sensors still provided a respectable degree of accuracy in detecting the specific tasks. Further, this model of HAR could be applied to other sports to more accurately assess exposures and thus better understand mechanisms of performance and musculoskeletal pain conditions.

## Supplementary information

**Additional file 1:** Detailed convolutional neural network model architecture.

## Data Availability

The datasets generated during and/or analysed during the current study are available from the corresponding author on reasonable request.

## Abbreviations

CNN: Convolutional neural network
HAR: Human activity recognition
L: Left
R: Right
