# Supplementary material for: Development of a Human Activity Recognition System for Ballet Tasks
Source: Sports Med Open. 2020 Feb 7;6:10. doi: 10.1186/s40798-020-0237-5 (PMC7007459; doi:10.1186/s40798-020-0237-5)
Supplement: Supplementary file 1 — Additional file 1: Detailed convolutional neural network model architecture. [file 40798_2020_237_MOESM1_ESM.docx]

INPUT

INPUT: 6 x wearable sensors graphs: triaxial accelerometer, gyroscope, magnetometer

FEATURE EXTRACTION LAYERS

LAYER 1 (20 FILTERS)

|  |  | **Window Size: 100 ms** | | | | | | | | | | | | | | | | | | | | |
| --- | --- | --- | --- | --- | --- | --- | --- | --- | --- | --- | --- | --- | --- | --- | --- | --- | --- | --- | --- | --- | --- | --- |
| L SHIN SENSOR | ACC | X |  |  |  |  |  |  |  |  |  |  |  |  |  |  |  |  |  |  |  |  |
|  |  | Y |  |  |  | Filter Size:  25 Horizontal  9 Vertical |  |  |  |  |  |  |  |  |  |  |  |  |  |  |  |  |
|  |  | Z |  |  |  |  |  |  |  |  |  |  |  |  |  |  |  |  |  |  |  |  |
|  | GYRO | X |  |  |  |  |  |  |  |  |  |  |  |  |  |  |  |  |  |  |  |  |
|  |  | Y |  |  |  |  |  |  |  |  |  |  |  |  |  |  |  |  |  |  |  |  |
|  |  | Z |  |  |  |  |  |  |  |  |  |  |  |  |  |  |  |  |  |  |  |  |
|  | MAG | X |  |  |  |  |  |  |  |  |  |  |  |  |  |  |  |  |  |  |  |  |
|  |  | Y |  |  |  |  |  |  |  |  |  |  |  |  |  |  |  |  |  |  |  |  |
|  |  | Z |  |  |  |  |  |  |  |  |  |  |  |  |  |  |  |  |  |  |  |  |

Stride Size: Horizontal 5ms, Vertical 9 outputs

Activation Function: Rectified Linear Unit

Pooling: Max Pool Layer

Data compressed by 5

100ms=20ms

LAYER 2 (20 FILTERS)

|  |  | **Window Size: 20 ms** | | | | |
| --- | --- | --- | --- | --- | --- | --- |
| L SHIN SENSOR | ACC | X | Filter Size:  10 Horizontal  9 Vertical |  |  |  |
|  |  | Y |  |  |  |  |
|  |  | Z |  |  |  |  |
|  | GYRO | X |  |  |  |  |
|  |  | Y |  |  |  |  |
|  |  | Z |  |  |  |  |
|  | MAG | X |  |  |  |  |
|  |  | Y |  |  |  |  |
|  |  | Z |  |  |  |  |

Stride Size: Horizontal 1ms, Vertical 9 outputs

Activation Function: Rectified Linear Unit

Pooling: Max Pool Layer

Data compressed by 4

20ms=5ms

CLASSIFICATION LAYERS

LAYER 3

Fully Connected Layer (50 neurons)

Activation function: Rectified Linear Unit

Fully Connected Layer (# of ballet movements- dependent upon level of classification)

Activation Function: Categorical Cross Entropy

Fully Connected Layer (10 neurons)

Activation function: Rectified Linear Unit

LAYER 4

Definitions of terms

| Stride | The size of the step that the filter takes in the convolution layer |
| --- | --- |
| Rectified Linear Unit | A non-linear activation function to transform input values = max(0, x) |
| Pooling and Max Pool Layer | A function that reduces the spatial representation (size) of the data in a neural network. This helps reduce the amount of parameters and computation in addition to reducing overfitting when training the network. |
| Neuron | A mathematical approximation of a biological neuron. It takes a vector of inputs, performs a transformation, and outputs a single scalar value. |
| Fully Connected Layer | Neurons in a fully connected layer have neurons (nodes) connected to all activations in the previous layer. |
| Categorical Cross Entropy | A loss (error) function used to evaluate how well a model performs on a multi-class classification task. |
